# Smoking-Induced Inhibition of Number and Activity of Endothelial Progenitor Cells and Nitric Oxide in Males Were Reversed by Estradiol in Premenopausal Females

**DOI:** 10.1155/2020/9352518

**Published:** 2020-05-12

**Authors:** Yijia Shao, Liang Luo, Zi Ren, Jiayi Guo, Xingxing Xiao, Jiana Huang, Haitao Zeng, Hong Zhan

**Affiliations:** ^1^Department of Hypertension and Vascular Disease, The First Affiliated Hospital of Sun Yat-Sen University, Guangzhou 510080, China; ^2^NHC Key Laboratory of Assisted Circulation (Sun Yat-Sen University), Guangzhou, Guangdong 510080, China; ^3^Department of Critical Care Medicine, The Seventh Affiliated Hospital of Sun Yat-Sen University, ShenZhen, Guangdong 518107, China; ^4^Center for Reproductive Medicine, Sixth Affiliated Hospital, Sun Yat-Sen University, Guangzhou 510605, China; ^5^Division of Emergency Medicine, Department of Emergency Intensive Care Unit, The First Affiliated Hospital, Sun Yat-Sen University, Guangzhou 510080, China

## Abstract

**Objectives:**

The number and activity of circulating EPCs were enhanced in premenopausal women contrast to postmenopausal females and age-matched males. Here, we investigated whether this favorable effect exists in premenopausal women and age-matched men with cigarette smoking.

**Methods:**

In a cross-sectional study, the number and activity of circulating EPCs and nitric oxide production (NO) as well as flow-mediated vasodilation (FMD) in both premenopausal women and age-matched men with or without cigarette smoking were studied.

**Results:**

Compared with age-matched men with or without smoking, the number and function of circulating EPCs as well as NO level in premenopausal women were obviously higher than that in the former and not affected by smoking. The number and function of circulating EPCs as well as NO level in male smokers were shown to be the most strongly inhibited. Furthermore, there was significant correlation between EPC number and activity, plasma NO level, and NO secretion by EPCs and FMD.

**Conclusions:**

Estradiol was deemed to play an important role in enhancing the number and activity of EPCs and NO production in premenopausal women even when affected by smoking, which may be the important mechanisms underlying vascular protection of estradiol in premenopausal women, but not in age-matched men.

## 1. Introduction

Cardiovascular disease (CVD) is the leading cause of deaths among both men and women globally [1]. However, sexual dimorphism exists in the incidence of CVD with a phenomenon that premenopausal women tend to have a lower prevalence, but menopausal females tend to have a higher prevalence compared to age-matched males [2]. It is widely believed that the differences in sex hormones, especially female estrogens, may partly account for this favorable phenomenon [3], but the possible mechanisms of the cardiovascular protections associating with premenopausal women are yet to be explored.

Endothelial progenitor cells (EPCs) are originated from bone marrow under various physiological or pathological conditions and then circulating in the peripheral blood, involving in the process of endothelial repairing by adhering to the inner wall of injured blood vessels and differentiating into mature endothelial cells [4, 5]. Furthermore, it has been discovered that levels of EPCs may be an important predictor of vascular function and cardiovascular incidence and cardiac deaths [6, 7]. Our previous studies have demonstrated that the number and activity of circulating EPCs were reduced in elderly men, coronary artery disease, and essential hypertension [8–10]. Investigations revealed that the number and activity of circulating EPCs were enhanced in premenopausal women contrast to postmenopausal females and age-matched men [11, 12]. Our results concur with these previous studies in which the number and activity of circulating EPCs were preserved in prehypertensive premenopausal women due to the restoration of nitric oxide (NO) production [13]. The situation changes; however, when prehypertensive premenopausal women combined with diabetes mellitus, the number and activity of circulating EPCs were predominantly hampered [14].

Cigarette smoking is one of most important risk factors for cardiovascular disease, and after 5 years of smoking cessation, CVD risk obviously declined but still remained higher than that in never smokers [15]. Unfortunately, the number of female smokers is increasing significantly [16]. It has reported that the number of circulating EPCs was reduced in chronic smokers and this makes smokers more vulnerable to CVD [17]. However, whether the detrimental effect still exists in young female smokers is not clear. Accordingly, we evaluated the numbers and activity of circulating EPCs as well as flow-mediated vasodilation (FMD) in both premenopausal women and age-matched men with or without cigarette smoking. The present study will extend our knowledge of the effects of cigarette smoking on EPCs and FMD in premenopausal women, which may shed some light on the mechanisms behind the cardioprotective effects particularly possessed in young women, especially those with CAD risk factors.

## 2. Materials and Methods

### 2.1. Study Details and Inclusion and Exclusion Criteria

Eighty healthy volunteers (female : male = 1 : 1) aged between 18 and 50 years old from the community were enrolled in the study and divided into four groups: female smoker, female nonsmoker, male smoker, and male nonsmoker. All the women in the study were in normal menstrual state. Detailed medical history and both physical and laboratory examination were taken in all volunteers and subjects with CVD, diabetes, hyperlipidemia, infectious disease, and severe trauma and receiving operation in early last month were excluded. This study was approved by the Sixth Affiliated Hospital of Sun Yat-sen University Ethics Review Board. Informed consent was obtained from all subjects enrolled in this study. The clinical characteristics of the population studied are summarized in Table 1.

### 2.2. The Count of Circulating EPCs by Flow Cytometry Analysis and Cell Culture Assay

Detection of EPCs was performed as in our previous studies [10, 13, 14]. Flow cytometry analysis was performed according to the protocol, and the count of circulating EPCs was determined by the ratio of CD34 + KDR + cells per 100 peripheral blood mononuclear cells (PBMNCs).

The circulating EPCs were isolated and cultured in vitro and then quantified by determining the uptake of 1,1′-dioctadecyl-3,3,3′,3′-tetramethylindo-carbocyanine perchlorate-labeled acetylated LDL (DiI-acLDL) and the staining of FITC-labeled Ulex europeus agglutin (lectin).

### 2.3. The Migration and Proliferation of EPCs

Migration and proliferation assays were performed in our previous studies [10, 13, 14]. EPC migration was determined using a modified Boyden chamber. Briefly, 2 × 10^4^ EPCs were placed in the upper chamber and the whole chamber was incubated in EBM-containing human recombinant VEGF (50 ng/mL) at 37°C for 24 h. Afterwards, the lower side of the filter was fixed with 2% paraformaldehyde and stained with DAPI for cell nuclei and then counted manually in 3 random microscopic fields.

EPC proliferation was determined by the MTT method in accordance with the protocol. After 24 h of serum-free pretreatment, EPCs were supplemented with 10 *μ*l MTT (Fluka Co. Product) and incubated for another 4 h, and then the EPC preparation was shaken with DMSO and the OD value was measured at 490 nm.

### 2.4. Measurement of NO, VEGF, and GM-CSF Levels from Plasma and EPCs Secretion

Nitrite, the stable metabolite of NO, was measured using the Greiss method as described previously [18], in which the total NO was determined based on the enzymatic conversion of NO3− to NO2− by nitrate reductase and detection of nitrite as an azo dye product of the Greiss reaction. Levels of VEGF and GM-CSF were measured by highly sensitive ELISA assays (R&D Systems, Wiesbaden, Germany) in accordance with our previous studies.

### 2.5. Measurement of Flow-Mediated Vasodilation in the Brachial Artery

For evaluation of endothelial function in subjects, flow-mediated vasodilation measurement in the brachial artery was performed as we described previously [14]. The brachial artery diameter was imaged with a 5–12 MHz linear array transducer ultrasound system at a location 3 to 7 cm above the right elbow, and the diameters at baseline (D0) and after reactive hyperemia (D1) and sublingual nitroglycerine (D2) were recorded. The flow-mediated vasodilation [(D1−D0)/D0×100%] was regarded as endothelium-dependent vasodilation. The nitroglycerine-mediated vasodilatation [(D2 − D0)/D0 × 100%] was regarded as endothelium-independent vasodilatation. The repeatability coefficients of flow-mediated vasodilation and nitroglycerine-mediated vasodilation on the same person in a 2-day interval were 0.93 and 0.91, respectively.

### 2.6. Statistical Analysis

Data were presented as mean ± SD. Statistical analysis was performed with SPSS 23.0 software for Windows (SPSS Software, Chicago, IL). Comparisons among the four groups were analyzed by two-factor analysis of variance. Univariate correlations were calculated using Pearson's coefficient (*r*). *P* < 0.05 was considered statistically significant.

## 3. Results

### 3.1. Baseline Characteristics

All healthy volunteers who participated in the study were included and excluded according to the methodology of the abovementioned trial design. As shown in Table 1, the four groups were similar in terms of age, body mass index, systolic blood pressure, diastolic blood pressure, and heart rate. There were no differences between the levels of AST, ALT, BUN, creatinine, LDL, HDL, total cholesterol, triglyceride, and fasting plasma glucose. Compared with men, the height and weight were lower in premenopausal women, in the condition of smoking or not (*P* < 0.05). Estradiol was higher and flow-mediated vasodilation was better in premenopausal women than that in men with or without smoking (*P* < 0.05).

### 3.2. Effect of Smoking on the Gender-Related Decline in the Number of Circulating EPCs

**Figure 1:**
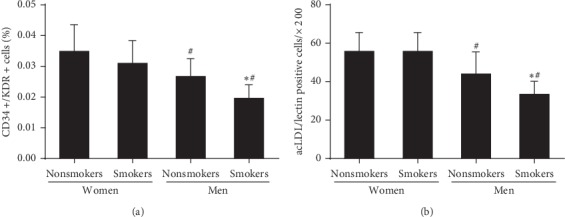
The number of circulating EPCs in the four groups, evaluated by (a) FACS analysis and using (b) phase-contrast fluorescent microscope; the number of circulating EPCs in male nonsmokers and smokers was lower than those in female premenopausal nonsmokers and smokers. The EPC number in male smokers was lower than that in female premenopausal smokers. However, no significant difference in the level of number of circulating EPCs between female premenopausal nonsmokers and smokers was found. Data are given as mean ± SD. ^*∗*^Vs nonsmokers; ^*#*^vs premenopausal women.

As shown in Figure 1, the number of circulating EPCs characterized by FACS analysis or fluorescence staining in male with or without smoking was lower than that in premenopausal female (*P* < 0.05). Compared with male nonsmokers, the number of circulating EPCs was further lower in male smokers (*P* < 0.05). However, the variance of EPC level related to smoking was disappeared in premenopausal women.

### 3.3. Effect of Smoking on the Gender-Related Decline in the Activity of Circulating EPCs

**Figure 2:**
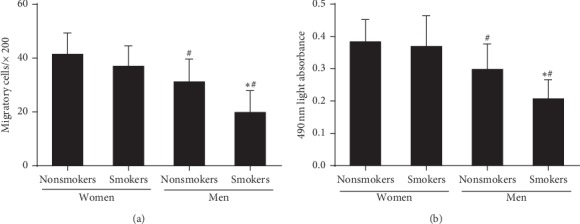
The activity of circulating EPCs in the four groups. The migratory (a) and proliferative (b) activities of circulating EPCs in male nonsmokers and smokers were lower than those in female premenopausal nonsmokers and smokers. There was no difference in the migratory (a) and proliferative (b) activity between female premenopausal nonsmokers and smokers. Nevertheless, the EPC function in male smokers was lower than that in male nonsmokers. Data are given as mean ± SD. ^*∗*^Vs nonsmokers; ^*#*^vs premenopausal women.

As shown in Figure 2, the migration and proliferation of circulating EPCs in male with or without smoking was lower than that in premenopausal female (*P* < 0.05). Compared with male nonsmokers, the migration and proliferation of circulating EPCs were further lower in male smokers (*P* < 0.05). However, the variance of EPC function related to smoking was disappeared in premenopausal women.

### 3.4. Effect of Smoking on the Gender-Related Decline in the Plasma NO Level

**Figure 3:**
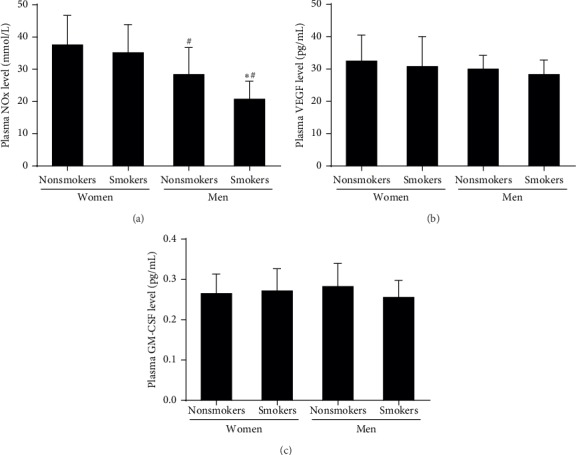
The plasma NO, VEGF, and GM-CSF levels in the four groups. (a) The plasma NO level in male nonsmokers and smokers was lower than that in female premenopausal nonsmokers and smokers. No difference in plasma NO level between female nonsmokers and smokers was found. The plasma NO level in male smokers was lower than that in male smokers. (b) There was no significant difference in the plasma VEGF level between the four groups. (c) There was no significant difference in the plasma GM-CSF level between the four groups. Data are given as mean ± SD. ^*∗*^vs nonsmokers; ^*#*^vs premenopausal women.

As shown in Figure 3, the plasma NO level in male with or without smoking was lower than that in premenopausal female (*P* < 0.05). Compared with male nonsmokers, the plasma NO level was further lower in male smokers (*P* < 0.05). However, the VEGF and GM-CSF levels were related to neither gender nor smoking.

### 3.5. Effect of Smoking on the Gender-Related Decline in the NO Secretion by EPCs

**Figure 4:**
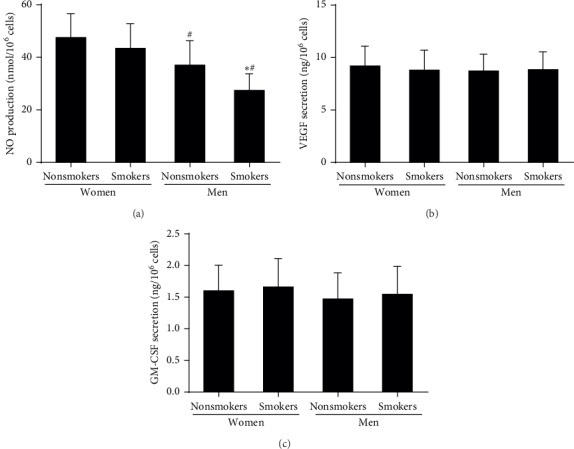
The NO, VEGF, and GM-CSF secretion by EPCs in the four groups. (a) The NO secretion by EPCs in male nonsmokers and smokers was lower than that in female premenopausal nonsmokers and smokers. No difference in NO secretion by EPCs between female premenopausal nonsmokers and smokers was found. However, the plasma NO level in male smokers was lower than that in male smokers. (b) There was no significant difference in VEGF secretion by EPCs between the four groups. (c) There was no significant difference in GM-CSF secretion by EPCs between the four groups. Data are given as mean ± SD. ^*∗*^Vs nonsmokers; ^*#*^vs premenopausal women.

As shown in Figure 4, the NO secreted by EPCs in male with or without smoking was less than that in premenopausal female (*P* < 0.05). Compared with male nonsmokers, the NO secreted by EPCs was further less in male smokers (*P* < 0.05). However, the VEGF and GM-CSF secreted by EPCs were related to neither gender nor smoking.

### 3.6. Correlation between Circulating EPCs or NO Level and FMD

**Figure 5:**
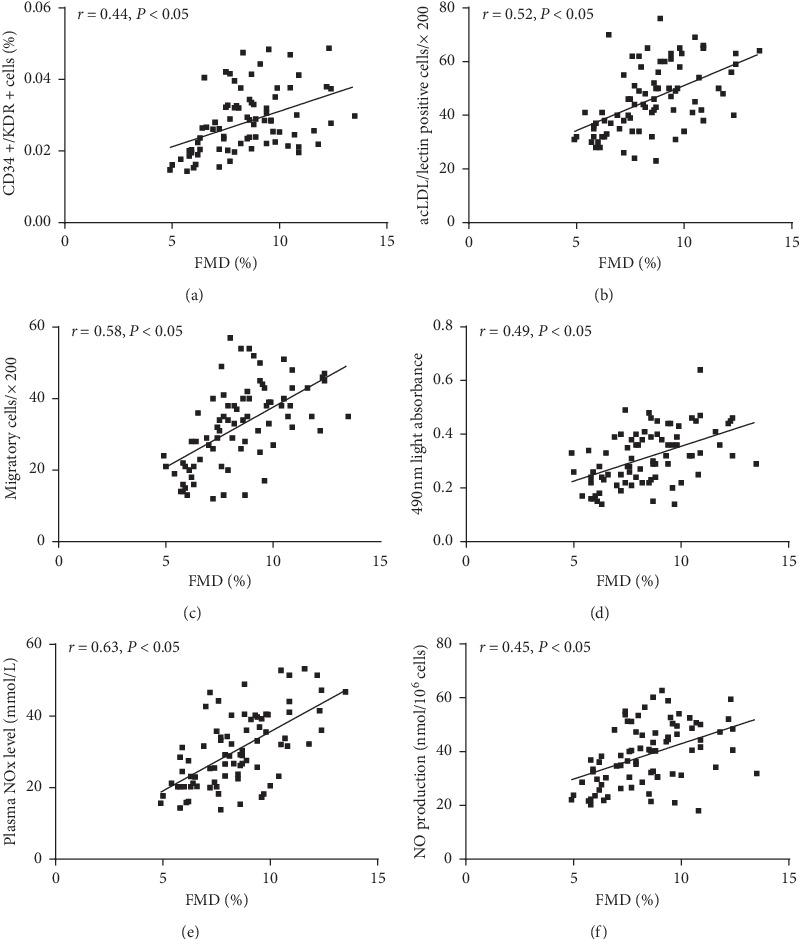
The correlation between circulating EPCs or NO level and FMD. The number of circulating EPCs evaluated by FACS (a) or by cell culture (b) correlated with the FMD. There was a correlation between the proliferatory (c) or migratory EPCs (d) and FMD. In addition, there was a correlation between the plasma NO level (e) or NO secretion by EPCs (f) and FMD.

As shown in Figure 5, there was a significant correlation between the FMD and the number of circulating EPCs evaluated by FACS (*r* = 0.44, *P* < 0.05) or by cell culture (*r* = 0.52, *P* < 0.05). Similarly, there was a significant correlation between the FMD and the migration (*r* = 0.58, *P* < 0.05) or proliferation (*r* = 0.49, *P* < 0.05) of circulating EPCs. We also found that the plasma NO level (*r* = 0.63, *P* < 0.05) or NO secretion (*r* = 0.45, *P* < 0.05) by EPCs significantly correlated with the FMD.

## 4. Discussion

Our present study indicated that the number and function of circulating EPCs were enhanced in premenopausal women contrast to age-matched men, which was consistent with other previous research [11, 12]. As an aside, it is interesting to note that the number and function of circulating EPCs were further attenuated in male smokers; however, the impairments of EPC number and function caused by smoking were disappeared in premenopausal women. Cigarette smoking is one of most important risk factors for cardiovascular CVD and which could be attributed to the inhibition of circulating EPCs in chronic smokers [15, 17]. However, the detrimental effect of smoking on EPCs did not exist in premenopausal female. As mentioned above, the level of estradiol was significantly higher in premenopausal women than that in men with or without smoking; in view of the comparable clinical characteristics among the four groups, it is reasonable to infer that estradiol may account for the higher degree of EPC number and function in premenopausal women and the protection against unfavorable effect of smoking on EPCs. It is widely recognized that female estrogens exhibit protective effects on the cardiovascular system in different ways [3], and our findings demonstrated that estradiol-induced enhancements of EPC number and function could be one of the pathways, whether there was smoking or not.

NO, VEGF, and GM-CSF play important roles in the regulation of the number and function of circulating EPCs [18–21]. Furthermore, many studies indicated that smoking was associated with the generation and activity of NO, VEGF, and GM-CSF [22–24]. Results obtained in our research demonstrated that NO level in male with or without smoking was lower than that in premenopausal female, whatever in plasma or cell supernatant secreted by cultured EPC, and compared with male nonsmokers, NO level was further lower in male smokers. However, the VEGF and GM-CSF levels were related to neither gender nor smoking. The changes of NO were consistent with the variances of EPC number and function, which provided a clue that decline in the count and activity of circulating EPCs may attribute to the inhibition of NO production. NO has been shown to be an important signaling molecule to accommodate functional homeostasis of EPCs. Insufficient NOS/NO/MMP9 pathway resulted in impaired mobilization of EPCs in hypertension [25]. In patients with diabetes mellitus, altered eNOS activity led to the suppression of EPC mobilization and function, which seems to contribute to the pathogenesis of vascular disease in diabetes [26]. Even in healthy subjects, acute exercise-induced NO production appeared to be strictly related to the upregulation of circulating EPCs [18].

A lot of attention has been paid to the intriguing phenomenon that premenopausal women have a lower prevalence of CVD until into menopause, and after that, the incidence surges and may eventually surpass that of men [27]. It was generally accepted that the estrogens play a key role in maintaining of the lower rates of CVD in premenopausal women [3]. The production of NO has been recognized as the most important process which mediates the vascular protection of estradiol [28]. It can thus be inferred that the higher level of estradiol in premenopausal women in the presence of smoking or not prompted the production of NO and thus enhanced the number and activity of circulating EPCs. However, in age-matched males who have a low-level of estradiol, the production of NO and the EPC number and activity were suppressed, especially when affected by tobacco. This result was supported by a previous study in which smoking was associated with reduced NO generation and eNOS activity [24].

As a manifestation of endothelial function, impaired FMD was supposed to be one of the earliest adverse effects of cigarette smoking on vascular function, and smoking-induced reduced NO production may probably be responsible for the downregulation of FMD [29, 30]. Here, we found that not only EPC number and activity correlated with FMD but also the plasma NO level and NO secretion by EPCs correlated with FMD. The study backed up this idea that reduced NO production may suppress the number and activity of EPCs and ultimately lead to endothelial dysfunction in men, especially when affected with tobacco. This suggested that estrogen may play a role in reversing the impairments of smoking on EPCs and vascular homeostasis. These results have intensified our understandings of the noxious effects of smoking and the favorable effects of estradiol on CVD and the balance between these two conflicting factors would determine the degree of the alteration of EPCs and endothelial function.

However, it should be noted that the present study has the limitation of lacking detailed molecular pathways to elaborate the mechanisms underlying the alteration of number and activity of EPCs and NO production among the four groups.

## 5. Conclusions

To our knowledge, this was the first time to demonstrate that estradiol may exert vital roles in enhancing the number and activity of EPCs and NO production in premenopausal women even in the presence of smoking, which may be the important mechanism underlying vascular protection of estradiol. Our findings provided a new insight into the vascular protection of high-level estradiol in premenopausal women even in the presence of smoking, and the number and activity of EPCs and NO production may be responsible for the alteration of vascular function.

## Figures and Tables

**Table 1:** Clinical and biochemical characteristics of four groups.

Characteristics	Female nonsmokers (*n* = 20)	Female smokers (*n* = 20)	Male nonsmokers (*n* = 20)	Male smokers (*n* = 20)
Age (years)	42.8 ± 3.6	43.4 ± 3.9	44.6 ± 3.7	43.6 ± 3.8
Smoking (pack-years)	NA	14.8 ± 4.5	NA	16.6 ± 5.5
Height (cm)	162.3 ± 5.7	161.9 ± 5.3	168.4 ± 4.9^#^	169.6 ± 6.6^#^
Weight (kg)	60.2 ± 5.2	58.7 ± 5.1	64.6 ± 6.2^#^	66.4 ± 5.0^#^
BMI (kg/cm^2^)	22.9 ± 2.3	22.4 ± 1.8	22.8 ± 2.2	23.1 ± 1.5
Systolic blood pressure (mmHg)	118.7 ± 10.0	121.5 ± 8.9	117.3 ± 9.6	120.6 ± 9.3
Diastolic blood pressure (mmHg)	73.3 ± 6.5	75.5 ± 7.4	72.8 ± 6.1	74.6 ± 7.0
Heart rate (beats/min)	73.2 ± 6.3	72.9 ± 7.8	74.4 ± 8.0	71.1 ± 7.7
AST (mmol/L)	23.6 ± 5.0	25.7 ± 6.0	26.5 ± 5.2	24.4 ± 5.7
ALT (mmol/L)	20.6 ± 4.8	23.6 ± 7.1	24.7 ± 5.6	22.2 ± 5.5
BUN (mmol/L)	4.6 ± 0.8	4.8 ± 0.9	4.9 ± 0.8	5.0 ± 0.7
Cr (mmol/L)	63.2 ± 13.2	65.3 ± 15.0	69.1 ± 14.7	68.3 ± 15.7
LDL (mmol/L)	2.68 ± 0.38	2.78 ± 0.41	2.58 ± 0.37	2.66 ± 0.30
TC (mmol/L)	4.65 ± 0.49	4.77 ± 0.52	4.48 ± 0.61	4.61 ± 0.47
HDL (mmol/L)	1.39 ± 0.24	1.35 ± 0.22	1.41 ± 0.18	1.43 ± 0.17
TG (mmol/L)	1.41 ± 0.20	1.45 ± 0.20	1.38 ± 0.18	1.36 ± 0.16
FPG (mmol/L)	4.81 ± 0.54	4.51 ± 0.50	4.40 ± 0.41	4.61 ± 0.53
Estradiol (pmol/L)	203.6 ± 20.6	198.3 ± 22.9	103.5 ± 15.0^#^	95.7 ± 11.0^#^
FMD (%)	9.72 ± 1.73	8.87 ± 1.67	8.34 ± 1.59^#^	6.89 ± 1.72^#∗^

BMI, body mass index; LDL, low-density lipoprotein; TC, total cholesterol; HDL, high density lipoprotein; TG, triglyceride; FPG, fasting plasma glucose; FMD, flow-mediated brachial artery dilatation. Smokers are defined as individuals with smoking ≥10 pack-year. Nonsmokers are defined as individuals who never smoked. Data are given as mean ± SD. ^*∗*^vs nonsmokers; ^#^vs premenopausal women.

## Data Availability

The data used to support the findings of this study are included within the tables of the article.

## References

[1] Naghavi M., Abajobir A. A., Abbafati C. (2017). Global, regional, and national age-sex specific mortality for 264 causes of death, 1980–2016: a systematic analysis for the global burden of disease study 2016. *The Lancet*.

[2] Pabbidi M. R., Kuppusamy M., Didion S. P., Sanapureddy P., Reed J. T., Sontakke S. P. (2018). Sex differences in the vascular function and related mechanisms: role of 17*β*-estradiol. *American Journal of Physiology-Heart and Circulatory Physiology*.

[3] Mendelsohn M. E., Karas R. H. (1999). The protective effects of estrogen on the cardiovascular system. *New England Journal of Medicine*.

[4] Shantsila E., Watson T., Lip G. Y. H. (2007). Endothelial progenitor cells in cardiovascular disorders. *Journal of the American College of Cardiology*.

[5] Asahara T., Murohara T., Sullivan A. (1997). Isolation of putative progenitor endothelial cells for angiogenesis. *Science*.

[6] Hill J. M., Zalos G., Halcox J. P. J. (2003). Circulating endothelial progenitor cells, vascular function, and cardiovascular risk. *New England Journal of Medicine*.

[7] Werner N., Kosiol S., Schiegl T. (2005). Circulating endothelial progenitor cells and cardiovascular outcomes. *The New England Journal of Medicine*.

[8] Xia W. H., Yang Z., Xu S. Y. (2012). Age-related decline in reendothelialization capacity of human endothelial progenitor cells is restored by shear stress. *Hypertension*.

[9] Cao Z., Tong X., Xia W. (2016). CXCR7/p-ERK-signaling is a novel target for therapeutic vasculogenesis in patients with coronary artery disease. *Plos One*.

[10] Yang Z., Chen L., Su C. (2010). Impaired endothelial progenitor cell activity is associated with reduced arterial elasticity in patients with essential hypertension. *Clinical and Experimental Hypertension*.

[11] Fadini G. P., de Kreutzenberg S., Albiero M. (2008). Gender differences in endothelial progenitor cells and cardiovascular risk profile. *Arteriosclerosis, Thrombosis, and Vascular Biology*.

[12] Bulut D., Albrecht N., Imöhl M. (2007). Hormonal status modulates circulating endothelial progenitor cells. *Clinical Research in Cardiology*.

[13] Zhen Y., Xiao S., Ren Z. (2015). Increased endothelial progenitor cells and nitric oxide in young prehypertensive women. *The Journal of Clinical Hypertension*.

[14] Zeng H., Jiang Y., Tang H. (2016). Abnormal phosphorylation of Tie2/Akt/eNOS signaling pathway and decreased number or function of circulating endothelial progenitor cells in prehypertensive premenopausal women with diabetes mellitus. *BMC Endocrine Disorders*.

[15] Duncan M. S., Freiberg M. S., Greevy R. A., Kundu S., Vasan R. S., Tindle H. A. (2019). Association of smoking cessation with subsequent risk of cardiovascular disease. *JAMA*.

[16] Huxley R. R., Woodward M. (2011). Cigarette smoking as a risk factor for coronary heart disease in women compared with men: a systematic review and meta-analysis of prospective cohort studies. *The Lancet*.

[17] Kondo T., Hayashi M., Takeshita K. (2004). Smoking cessation rapidly increases circulating progenitor cells in peripheral blood in chronic smokers. *Arteriosclerosis, Thrombosis, and Vascular Biology*.

[18] Yang Z., Wang J.-M., Chen L., Luo C.-F., Tang A.-L., Tao J. (2007). Acute exercise-induced nitric oxide production contributes to upregulation of circulating endothelial progenitor cells in healthy subjects. *Journal of Human Hypertension*.

[19] Bonafè F., Guarnieri C., Muscari C. (2015). Nitric oxide regulates multiple functions and fate of adult progenitor and stem cells. *Journal of Physiology and Biochemistry*.

[20] Shurygin M. G., Shurygina I. A., Dremina N. N., Kanya O. V. (2015). Endogenous progenitors as the source of cell material for ischemic damage repair in experimental myocardial infarction under conditions of changed concentration of vascular endothelial growth factor. *Bulletin of Experimental Biology and Medicine*.

[21] Xue J., Du G., Shi J. (2014). Combined treatment with erythropoietin and granulocyte colony-stimulating factor enhances neovascularization and improves cardiac function after myocardial infarction. *Chinese Medical Journal*.

[22] Edirisinghe I., Yang S.-R., Yao H. (2008). VEGFR-2 inhibition augments cigarette smoke-induced oxidative stress and inflammatory responses leading to endothelial dysfunction. *The FASEB Journal*.

[23] Vlahos R., Bozinovski S., Chan S. P. J. (2010). Neutralizing granulocyte/macrophage colony-stimulating factor inhibits cigarette smoke-induced lung inflammation. *American Journal of Respiratory and Critical Care Medicine*.

[24] Barua R. S., Ambrose J. A., Eales-Reynolds L.-J., De Voe M. C., Zervas J. G., Saha D. C. (2001). Dysfunctional endothelial nitric oxide biosynthesis in healthy smokers with impaired endothelium-dependent vasodilatation. *Circulation*.

[25] Aleksinskaya M. A., van Faassen E. E. H., Nelissen J. (2013). Identification of free nitric oxide radicals in rat bone marrow: implications for progenitor cell mobilization in hypertension. *Plos One*.

[26] Thum T., Fraccarollo D., Schultheiss M. (2007). Endothelial nitric oxide synthase uncoupling impairs endothelial progenitor cell mobilization and function in diabetes. *Diabetes*.

[27] Reckelhoff J. F., Fortepiani L. A. (2004). Novel mechanisms responsible for postmenopausal hypertension. *Hypertension*.

[28] Stanhewicz A. E., Wenner M. M., Stachenfeld N. S. (2018). Sex differences in endothelial function important to vascular health and overall cardiovascular disease risk across the lifespan. *American Journal of Physiology-Heart and Circulatory Physiology*.

[29] Kiowski W., Linder L., Stoschitzky K. (1994). Diminished vascular response to inhibition of endothelium-derived nitric oxide and enhanced vasoconstriction to exogenously administered endothelin-1 in clinically healthy smokers. *Circulation*.

[30] Mcveigh G. E., Lemay L., Morgan D., Cohn J. N. (1996). Effects of long-term cigarette smoking on endothelium-dependent responses in humans. *The American Journal of Cardiology*.

